# Electroporation-Based Treatments in Urology

**DOI:** 10.3390/cancers12082208

**Published:** 2020-08-07

**Authors:** Aleksander Kiełbik, Wojciech Szlasa, Jolanta Saczko, Julita Kulbacka

**Affiliations:** 1 Faculty of Medicine, Wroclaw Medical University, 50-367 Wroclaw, Poland; aleksander.kielbik@outlook.com (A.K.); wojciech.szlasa@outlook.com (W.S.); 2 Department of Molecular and Cellular Biology, Wroclaw Medical University, 50-556 Wroclaw, Poland; jolanta.saczko@umed.wroc.pl

**Keywords:** ablation techniques, electrochemotherapy, electroporation, gene therapy, prostate cancer

## Abstract

The observation that an application of a pulsed electric field (PEF) resulted in an increased permeability of the cell membrane has led to the discovery of the phenomenon called electroporation (EP). Depending on the parameters of the electric current and cell features, electroporation can be either reversible or irreversible. The irreversible electroporation (IRE) found its use in urology as a non-thermal ablative method of prostate and renal cancer. As its mechanism is based on the permeabilization of cell membrane phospholipids, IRE (as well as other treatments based on EP) provides selectivity sparing extracellular proteins and matrix. Reversible EP enables the transfer of genes, drugs, and small exogenous proteins. In clinical practice, reversible EP can locally increase the uptake of cytotoxic drugs such as cisplatin and bleomycin. This approach is known as electrochemotherapy (ECT). Few in vivo and in vitro trials of ECT have been performed on urological cancers. EP provides the possibility of transmission of genes across the cell membrane. As the protocols of gene electrotransfer (GET) over the last few years have improved, EP has become a well-known technique for non-viral cell transfection. GET involves DNA transfection directly to the cancer or the host skin and muscle tissue. Among urological cancers, the GET of several plasmids encoding prostate cancer antigens has been investigated in clinical trials. This review brings into discussion the underlying mechanism of EP and an overview of the latest progress and development perspectives of EP-based treatments in urology.

## 1. Introduction

The prevalence of kidney, prostate, and bladder cancer is increasing rapidly within the context of an ageing population [1]. Urological cancers are generally regarded as a problem predominantly concerning wealthier countries. However, risk factors such as tobacco smoking, diet, and lifestyle inevitably lead to increased prevalence among lower-income populations [1]. Easily available, safe, and economical therapies must be developed to reduce the degree of inequality in terms of incident cases and deaths due to urological cancers. The technique known as electroporation provides the possibility for efficient focal ablation, intracellular delivery of cytostatics or other molecules such as calcium, and safe gene transfection. In its simplicity, no sophisticated equipment is required, except a pulse generator and an electrode.

The present development of sensitive methods of imaging, such as multiparametric magnetic resonance imaging (mpMRI), enables precise tumor localization, biopsy guidance, and focal therapy [2]. It is estimated that one-third of patients with mpMRI-detected and biopsy-proven lesions in the prostate gland are potential candidates for focal treatment [3]. For this group of patients, methods based on electroporation, irreversible electroporation (IRE), or electrochemotherapy (ECT) constitute possible choices of treatment.

With the increasing importance of immune therapy, new gene delivery techniques are being developed at a very fast pace. Among viral and non-viral approaches, gene electrotransfer (GET) is characterized by a high safety profile, acceptable efficiency, availability, and ease of application [4]. In vivo trials of anti-tumor therapy for urological cancers have already proven their efficacy.

There are few publications dedicated to physicians describing the more detailed mechanism of electroporation and the possibilities of its applications in urology. It should not be neglected that a few vulnerabilities of electroporation (EP) still require new solutions and need to be optimized. The underlying mechanisms should be discussed more frequently to increase awareness of physicians applying this method in the clinic and to illustrate its potential for future development.

## 2. The Theoretical Background of EP

Physiologically all human cells possess a resting voltage on their plasma membrane ranging from −70 mV to −30 mV, which is generally provided by Na^+^ and K^+^ active and passive transport through the membrane [5]. The foundation of the EP was the observation that cells exposed to an external electric field change the properties of their membranes and become more permeable [6,7]. Once the cell is exposed to an external electric field, an additional component of the voltage across the membrane occurs [8]. The induced transmembrane voltage for a spherical cell with a nonconductive membrane can be calculated with Laplace’s Equation (1):

(1) $$\Delta \Phi m=\frac{3}{2}ER\cos\theta$$

where *E* is the electric field in the region of a cell, R is cell radius, and *θ* is the angle measured from the center of the cell to the direction of the field [8].

When the transmembrane potential reaches 200–250 mV, parts of the membrane become highly permeable [9,10]. Permeabilization is a local process, and the fraction of the permeabilized membrane strongly correlates with the electric field intensity [11,12]. The organization of the membranes permeable spots is inhomogeneous [12].

To permeabilize the cell membrane, usually, a series of electrical rectangular pulses are applied [13]. Depending on the pulse duration, there could be distinguished nanosecond, microsecond, or millisecond electroporation protocols [14]. Today, microsecond pulses find clinical application in IRE for the ablation therapy of prostate cancer [15]. The use of microsecond electric pulse in IRE for other urological tumors, such as kidney cancer (NCT01967407, NCT02828709, NCT02298608) and urinary bladder neoplasm (NCT02430623), is continually being tested in clinical trials. Further considering urological tumors, nanosecond electric pulses are studied only at the in vitro level [16].

The precise molecular mechanism of EP is not fully understood. However, some properties of pulsed electric fields (PEFs), as well as cell features affecting the electropermeabilization, remain defined. The influence of the external electric field on the transmembrane voltage varies depending on the shape of the cells. The position and orientation of cells to the electric field also determines the transmembrane voltage [17]. The density of defects in the cell membrane depend on pulse duration and number of pulses [12,13,18].

The following three modalities of electric-field-aided treatment methods can be distinguished: irreversible electroporation, electrochemotherapy and gene electrotransfer. Each of them is based on the use of a pulsed electric field which induces changes in the membranes of the target cells, making them more permeable. However, each technique differs in mechanism of action, uses different parameters of the electric field, and has varied aims of application in the clinical setting. This will be continually expanded upon and described in the following sections.

The process of electroporation is complex and should not be simplified only to the phenomenon of permeabilizing the cell membrane. The electrical field affect cell permeability and also generates a transient mechanical force that stretches the spherical membrane [19]. EP may lead to cytoskeleton destabilization and changes in membrane elasticity [20]. It was observed that electrical pulses generate reactive oxygen species at the permeabilized loci [21]. However, Michel et al. proved that even if reactive oxygen species appear, their presence does not induce membrane permeabilization [22]. Electropermeabilization is followed by water flux and, consequently, by the osmotic swelling of cells [23]. When the cellular membrane becomes permeable, the leakage of metabolites such as ATP from the cytoplasm can be observed [24]. The intracellular ATP content is strongly related to the viability of the cells after electropermeabilization [25].

After permeabilization, the transfer of molecules such as drugs or even the insertion of exogenous proteins into the cell interior is possible [26,27]. The transfer of small molecules across the permeabilized membrane is driven by two factors—mainly by the concentration gradient across the membrane, but also by the post-pulse transmembrane potential [28]. EP enables the molecules to flow for seconds and up to a few minutes after the pulse [18]. The persistence of cells permeable state depends on the pulse duration and the number of pulses applied [29]. When applied PEFs do not overcome the irreversible electroporation threshold, the resealing of the cell membrane occurs [30,31,32]. The process engages multiple mechanisms. It was proven that cellular proteins and parallel the processes of endo and exocytosis contribute to cell membrane repair [33].

Generally, electroporation of tissue due to their inhomogeneity and anisotropy is difficult to foresee [34]. In electroporation, tissues should be considered as an insulator and conductor [35]. A few basic physical terms need to be reminded to clarify the dielectric properties of tissues. Permittivity is the measure of the capacitance of the material to store an electric field in the polarization of the medium. Conductivity is a measure of the ability of the material to conduct an electric current. Impedance is a measure of the opposition to the electric current in an electric circuit.

Conductivity and permittivity of tissues depend on the frequency of the applied electric pulses. Therefore, the tissue permittivity decreases in higher frequencies, and the conductivity increases. As the cell membrane becomes permeabilized, it increases its conductance [36]. The local changes in the electric field should be taken into account during individualized, patient-specific treatment planning [37]. It was observed that tumors are characterized by higher conductivity in comparison to normal tissues, probably due to regions of necrosis [38]. The phenomenon that the conductivity increases with electroporation enables it to effectively electroporate deeper structures of the tissue using lower voltages [39]. By measuring the electric conductivity changes in tissues, the cell permeabilization threshold can be estimated [40].

Controlling the real-time changes of the tissue impedance by the use of electrical impedance tomography (EIT) enables users to estimate the electroporated area [41]. Electrodes detect the changes in impedance caused by electropermeabilization. Subsequently, the obtained data is being transformed into the image of the electroporated area. Another technology—magnetic resonance electrical impedance tomography (MREIT)—combines EIT and magnetic resonance imaging (MRI). MREIT algorithms transfer the MRI image to digitally reconstruct the conductivity distribution inside the tissue. In contrast to EIT, it avoids the implementation of additional electrodes. In vivo and ex vivo research shows that MREIT can be applied in clinical electroporation-based procedures to improve the security of therapies [42,43]. In the future, we can expect the introduction of this technology in clinical settings.

As tissues are diverse and anisotropic, they possess distinct conduction properties [44]. Various tissues are characterized by a different proportion of the extracellular matrix, different water content, and irregular vascularization [35]. Inhomogeneity of conduction depends on the placement of the electrodes with respect to the major axis of tissue. If the tissue is organized in fibers, the detectable discrepancy of the longitudinal and transverse conductivity may occur [34]. In the longitudinal orientation of electrodes, the electricity is directed by cells organized in fibers, whereas in the transverse orientation, the charge has to overcome an extracellular matrix, which is less conductive than cells [35].

The pre-treatment computer simulation is helpful to plan the therapy, optimize pulse parameters, choose appropriate electrodes, and determinate their placement inside the tissue. Numerical modeling can be applied to predict the electric field distribution in inhomogeneous biological tissues. The simulation enables clinicians to visualize the electric field density in the targeted region and to determinate the range of irreversible and reversible electroporation and to determine the temperature rise occurring due to Joule heating [45].

## 3. IRE

**Figure 1:**
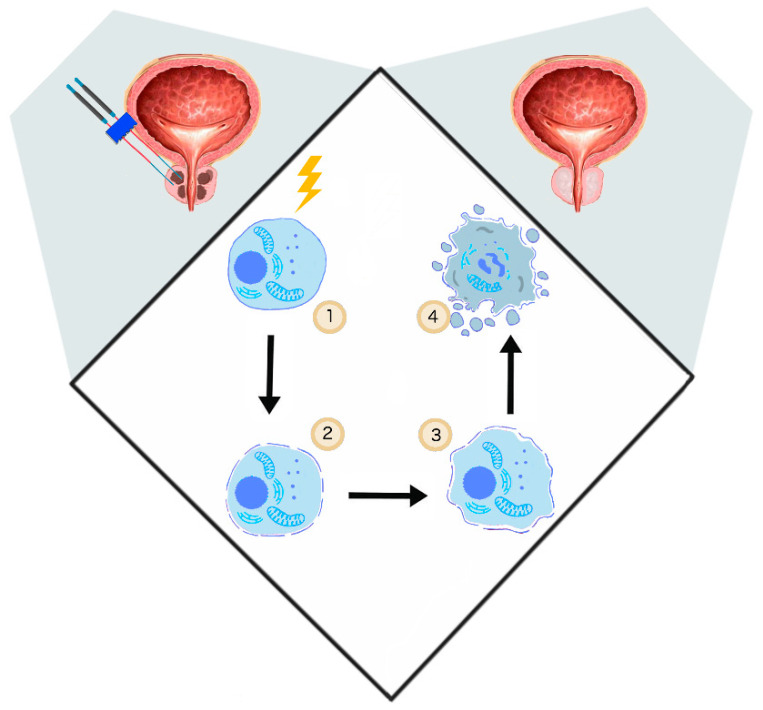
The general mechanism of irreversible electroporation (IRE). (1) Delivery of short electrical impulses (10–90 impulses 1000–2500 V/cm, 50–100 μs) [48], (2) cancer cell permeabilization, (3) irreversible permeabilization results in osmotic swelling [23], cytoskeleton destabilization [20], ATP depletion [24], (4) cell turns necrotic [46].

Davalos et al. showed that the application of PEFs promoting irreversible defects in the cellular lipid bilayer could be applied as a novel ablation method [46]. In contrast to other thermal tumor ablation possibilities such as microwave ablation, high-intensity focused ultrasound, or cryoablation, IRE is based on electropermeabilization and thus causes no excessive thermal effect [47] (Figure 1).

If the electric field strength is too far above the permeabilization threshold value, the permeabilized state is irreversible and results in cell destruction [46]. IRE does not require the application of chemotherapeutic agents. Several experiments investigated the specificity of IRE. It was shown that even if IRE has the potential to affect the nerves, the axonal regeneration process occurred two months after the procedure [49]. Another study on prostate gland ablation confirmed the preservation of the urethral wall, nerves, and vessels [15]. With optimal electric field parameters, IRE predominantly affects cell membrane phospholipids. Extracellular proteins, and the cell matrix are usually not affected [50].

Usually, the transmembrane potential of 1 V is sufficient to generate irreversible electroporation [46]. IRE is considered to be a non-thermal ablative method. However, the thermal effect occurs due to Joule heating and cannot be neglected. The amount of heat released is proportional to the electrode spacing and diameter and depends on repetition frequency [51]. Electrode configuration, the distance between electrodes, and the active tip length are the other factors that influence the IRE ablation zone. However, those features can be modulated during the procedure [52]. Due to the complexity of in vivo IRE procedure, pulse parameters have been established mainly experimentally. In in vivo studies, the electric field between 1000 V/cm and 2500 V/cm has been applied for IRE of the tumor. In most of the trials, the pulse durations ranged from 50 μs to 100 μs, and the pulse number varied between 10 and 90 [48].

Although IRE presents many advantages compared to other focal thermal ablation methods, there are a few issues that limit it in its clinical use. The successive ablation requires precise and parallel placement of multiple electrodes to optimize the ablation zone. IRE parameters should be personalized, since when adequately adjusted, can limit the damage caused by heat and extend the tissue ablation zone [46]. IRE is known to be minimally invasive. Nevertheless, it still requires general anesthesia and complete muscle relaxation [53]. Muscle contraction during the delivery of impulses can displace electrodes [54]. Moreover, to avoid cardiac arrhythmias, the electrical pulses need to be introduced during the refractory phase, and as a consequence, the electrode device should be synchronized with electrocardiography (ECG) [55].

In clinical practice, four different types of electrodes are used for IRE: needle, catheter, plate, and clamp. They can be applied percutaneously or intraoperatively. The endoscopic approaches for IRE ablations are currently still under development [56]. Furthermore, the endovascular IRE has been investigated for vascular smooth muscle cells. This minimally invasive method is used for creating a suitable niche for exogenous cell engraftment in regenerative surgery [57,58].

CT, MRI, and ultrasounds are often used for electroporation imaging. However, those methods cannot precisely estimate the electroporated area during the delivery of impulses [59,60,61]. The real-time imaging can be achieved by MREIT, which is a novel method was shortly described above.

### 3.1. IRE—Renal Cancer

Minimal invasive local ablation of renal cancer is an alternative treatment option for small tumors by patients who are not qualified or refuse to undergo surgery.

The effect of IRE on the porcine model was studied to investigate the histopathologic effect of renal cancer. Acute lesions, assessed 24 h after the procedure, were characterized by hemorrhagic necrosis. Three weeks after the procedure, tubules and glomeruli in the ablation zones were replaced by fibrous tissue. The extracellular matrix and transitional epithelium of the ablated pelvis generally remained intact, suggesting pelvic epithelium regeneration [62]. Urothelial regeneration was also observed in another study. MRI and intravenous urography confirmed the IRE sparring effect on renal calyxes, pelvis, and ureter [63].

The clinical study on patients with renal tumors confirmed the feasibility, safety, and efficacy of IRE as a focal ablation method [64,65]. The present clinical experience was summarized in Table 1. The procedure was performed percutaneously under general anesthesia and muscle relaxation. No significant changes in renal function were described after IRE performance. The mean procedural time was 2.1 h, including the need for general anesthesia; this might be considered as a disadvantage of IRE [64]. The post IRE imaging of the ablation zone showed similar characteristics to the marks observed after radiofrequency ablation and cryoablation [65].

The present European Association of Urology guidelines based on the low-quality study suggests a higher local recurrence rate for thermal ablation therapies compared to partial nephrectomy. However, simultaneously due to guidelines the focal therapy should be offered to frail and/or comorbid patients with small renal masses [67]. The new focal therapies, such as electroporation, should be developed to provide minimal-invasive treatment, suitable for comorbid patients, with the oncological outcome comparable to partial nephrectomy.

For renal cancer ablation techniques, such as radiofrequency ablation, cryoablation, and microwave ablation, are the most common techniques used [68]. However, due to the heatsink effect, their effectiveness is restricted once the targeted area is in the vicinity of blood vessels [69]. In contrary to mentioned methods, IRE is based on the permeabilization of the cell membrane, which results in cell death. It enables the treatment of tumors near critical structures with the preservation of healthy renal parenchyma and subsequent urothelial regeneration. However, to determine the oncological and functional outcome of IRE on renal cancer, more prospective studies with longer follow-up are required.

### 3.2. IRE—Prostate Cancer 

For prostate cancer treatment, IRE has been applied for focal therapy or, in case of a spreading tumor, for the whole gland ablation. Moreover, one published clinical trial concerns IRE for recurrent cancer after radiotherapy. The present experience from chosen studies, including the oncological and functional outcome, was summarized in Table 2. Due to expert consensus, patients with intermediate-risk and unifocal or multifocal prostate cancer are the potential candidates for focal treatment [70]. In the case of prostate cancer, IRE electrodes are usually transperineally inserted under the guidance of ultrasound [71].

Ablate and resect studies investigated the histopathological outcome of the therapy four weeks after the IRE procedure. The analysis showed that the ablation resulted in a sharply demarcated, necrotic, and fibrotic area of prostate tissue. The contralateral nerve bundles, as well as nerve bundles outside the ablation area, remained untouched. Urothelium impairment of prostatic urethra occurred in 9/16 patients after IRE. Short-term follow-ups have been unable to assess the possible recovery process [72].

The clinical study of IRE reported low-grade urinary complaints. The most frequent were painful micturition and occasional incontinence, thus pads were temporarily applied to the few patients that required them. The transperineal approach resulted in a small perineal swelling, inguinal lymphadenopathy, and temporarily swollen testis. However, pain in the lower abdomen occurred without evidence of a urinary tract infection. All complications emerged during the first week after the procedure, and nearly all disappeared between the first and fourth weeks after IRE [81]. In the largest study, only a mild decrease in sexual function was observed. Among patients who were potent before treatment, 76% (40/53) did not exhibit any change in erectile function [74].

After the treatment, cancer may progress due to in-field recurrence, when the tumor is present in the ablation zone, and out-of-field recurrence from the progression of contralateral prostate gland lesions. The largest prospective studies of IRE on prostate cancer confirm that the extension of treatment margin decreases the in-field recurrence rate. Twelve months after the procedure, the in-field recurrence appeared in 9.8% (10/102) of patients. However, among the group where treatment margins were extended to 10 mm, the in-field recurrence was 2.7% (2/74). Extended ablation did not show any significant impact on out-of-field recurrence (12.1%) (9/74) [74]. The study indicates that more precise identification of patients with multifocal disease is required to reduce out-of-field recurrence. That group would benefit from whole gland ablation. _68_Ga-PSMA PET fused with mpMR might provide the solution as it enables more accurate cancer detection [71].

The common danger for low-risk and arguably for intermediate-risk localized prostate cancer is overtreatment [82]. Prostatectomy and radiotherapy, although constantly developing, still involve serious risk of impotence and urine incontinence [83,84,85]. In careful risk stratification, better algorithms are required to determine the groups, which would benefit from focal treatment. With the development of imaging methods such as mpMRI, the use of minimal invasive focal treatments in prostate treatment will increase. Among the available approaches, high-intensity focused ultrasounds, cryotherapeutic ablation, or photodynamic therapy are the most investigated, but the data is still not sufficient to consider them as the first-line therapy. Today, electroporation for prostate cancer treatment is mainly offered within a clinical trial setting, but the therapy is rapidly developing, and treatment protocols are being enhanced. IRE, compared to other thermal ablative methods, may provide better preservation and regeneration possibilities of the urethra and neurovascular bundle. However, present studies cannot confirm this. No long-term follow-up in published studies preclude the evaluation of the exact oncological outcome. Other major deficits in the literature are the lack of the prospective randomized trial comparing IRE for prostate cancer treatment to other longer and better-investigated techniques such as high-intensity focused ultrasounds, cryotherapeutic ablation, photodynamic therapy, or radical prostatectomy. Nevertheless, for intermediate-risk prostate cancer, evaluation of the oncological outcome would require about 15 years of follow-up [71].

### 3.3. IRE—Urothelial Cancer

The development of a catheter electrode provides the possibility of intraluminal IRE. The feasibility study for the treatment of urothelial cancers was performed on the porcine model. The catheter-directed ablation showed the necrosis of urothelium, lamina propria, muscularis, and adventitia. However, the extracellular matrix of connective tissue remained untouched, preserving urethral integrity [86]. Short 28 day follow-up do not provide any information on the long-term situation [87]. One on-going clinical trial investigates the effect of IRE for unresectable urinary bladder cancer (NCT02430623).

### 3.4. High-Frequency Irreversible Electroporation

The improvement of selectivity can be provided by high frequent irreversible electroporation (H-FIRE), which uses short pulses (~1 μs). H-FIRE preferentially and proportionally to the size of the nucleus affects the cell interior. As large nuclei represent the typical feature of malignant cells, shorter pulses can more selectively target cancer cells [88]. It was also observed that H-FIRE triggers the local innate immune system and affects the tumor microenvironment promoting inflammation [89]. Moreover, H-FIRE does not cause muscle contraction. Prospectively, it might exclude intraoperative paralytics or cardiac synchronization, reducing the procedure time [90,91]. However, it was proved that H-FIRE pulses require a higher electric field to achieve the same level of cell permeabilization compared to standard monopolar pulses [91]. This phenomenon is called the cancellation effect and can be minimalized with an extension of pulse duration and interphase delay [92].

The first clinical study, which evaluated the possibility of prostate tumor ablation with H-FIRE, proved the safety for focal cancer treatment. Although several publications deny that H-FIRE causes muscle contraction, low-dose muscle relaxants were administrated before electroporation. Few patients underwent prostatectomy four weeks after the treatment. The histopathological examination revealed a well-demarcated ablation zone. The urethra, located in the ablation zone, remained intact. The examination proved its structural integrity with no evidence of necrosis in the submucosa. Sexual function and continence were preserved by all patients [75].

### 3.5. Immunomodulatory Effect of IRE

The immunomodulatory effect of IRE was evaluated in multiple studies. However, most of the published experimental trials concern the pancreas tumor. The major effect of IRE is a decrease of inhibitory Treg cells in the tumor microenvironment [93,94]. This effect was also observed after the application of calcium electroporation [95]. Interestingly, Beitel-White et al. presented the technology which enables the detection of a shift in Treg cell population after IRE [96]. Namely, changes in conductivity correlate with Treg cell depletion. The acquired information might influence the decision about following therapy [96]. Other studies reported the additional decrease of myeloid-derived suppressor cells in the tumor microenvironment after the performance of IRE [97]. To stimulate the immune response, lymphocytes should penetrate to the ablated sites of the tumor, and simultaneously, dendritic cells should be able to migrate and present the captured tumor antigens. The performance of IRE results in an increase of activated PD-1+ T cells in patient blood samples. IRE succeeded in boosting already existing T cell response and in its induction de novo [94]. Among lymphocytes, CD8+ and CD4+ T-cells are the main agents in anti-tumor response triggered by IRE [98,99]. Few experiments evaluated the possibility of performing IRE together with immunotherapy. The combined performance of IRE and checkpoint blockade with TLR7 stimulation resulted in a four-fold increase in IFNγ- secreting CD8+ T cells [97]. Furthermore, in another study of IRE on a murine model, an additional PD1 blockade again intensified the selective tumor infiltration by CD8+ T cells significantly prolonging the survival of mice [100]. The immunomodulatory effect of IRE can be perceived as an advantage of IRE to other ablation methods. One study compares the immunogenic effect of IRE and cryoablation. The outcome shows an enhanced infiltration of macrophages and T cells into electroporated tumor sites. A similar effect was not observed after cryoablation [101]. Among urological malignancies, the immunomodulatory effect was investigated and confirmed on murine renal tumors [102]. Considering the fast progress of immunotherapy, the application of IRE to stimulate the immune response and subsequent immunotherapy is a very promising approach. As predicted by Palucka et al., all cancers will be eventually treated with checkpoint inhibitors [103]. The application of PEFs can be utilized to boost the immune system. Nevertheless, future trials should evaluate the effectiveness of IRE and immunotherapy on urological cancers. Subsequently, the next step would be a well-designed prospective double-blind clinical study to fully confirm the efficiency of the therapy. Immunomodulatory effect of IRE raises the question of whether or not IRE could be performed before the surgery to stimulate the immune response. The study on the murine model showed the initial superiority of resection to IRE. However, after the repeated application of cancer cells, mice previously treated with IRE showed no recurrence, in contrast to mice after surgery. Furthermore, studies proved that IRE indeed triggers the vaccination effect and promotes the recognition of antigens by immune cells [97]. Beitel-White et al. suggest that surgery or other kinds of treatment in a multimodal approach should be performed at least 10 days after IRE to enable the activation of an adaptive immune response [96]. Based on the results of these studies, it is reasonable to combine IRE, surgery, and immunotherapy in future clinical trials [97,100].

## 4. Electrochemotherapy

**Figure 2:**
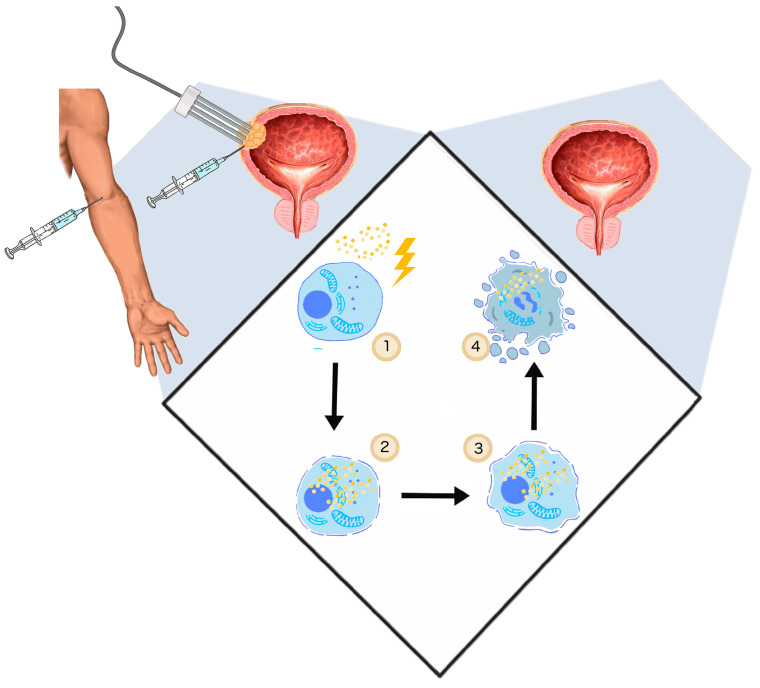
The general mechanism of ECT. (1) Two steps of ECT—administration of the drug (cisplatin, bleomycin) and application of PEFs (1000 V/cm, 100 μs, 9); (2) once the cell membrane gets permeabilized, the cytostatic drug penetrates to cell interior; (3) initiation of cell death by drug cytotoxicity, systemic anti-tumor reaction [108]; vasoconstriction, thus the ischemia of cancer cells [112] (4); and cell turns necrotic/apoptotic.

The application of the electric fields to enhance the intracellular anti-cancer drug uptake was studied and described by Mir et al. in 1991 [104]. Uptake of membrane non-permeable drugs can be locally increased when their intravenous or intratumoral administration is followed by electroporation. Clinically, in an approach commonly called electrochemotherapy, cisplatin and bleomycin are used [105]. Apart from cytostatics, intracellular uptake of calcium ions can be enhanced with electroporation as well. This novel solution showed no systemic toxicity and high efficiency in the in vitro and in vivo treatment of various cancer types [106]. In standard ECT, trains of eight 100-μs-long pulses are applied to achieve the reversible permeabilization of cells. The electroporation-mediated internalization of chemotherapeutics involves different mechanisms. During the application of PEFs, charged molecules cross the plasma membrane moved by electrophoretic forces. Subsequently, once the cell is in the permeable state, small hydrophilic molecules can enter the cell interior diffusing through the permeabilized area [107]. ECT anti-tumor effect cannot be explained only by increased uptake of cytostatics. Firstly, ECT showed high efficiency in in vivo studies as it stimulates the immune response. After the immunogenic death of electroporated cells, cancer antigens can be captured and recognized by dendritic cells and eventually increase antitumor response [108]. ECT could be considered as the adjuvant immunogen electrotransfer to peritumoral tissue [109]. The process leads to the local effects and triggers the systematic response against the metastases–abscopal effect [110]. Second, electroporation causes an increase in vessel permeability and constriction of arterioles resulting in so-called “vascular lock” [32]. The effect provides the targeted accumulation of the intravenously administrated drug [111] (Figure 2).

The data of ECT on urological cancers still remain restricted. However, with promising in vivo results, together with the technical progress of endoscopic or percutaneous approaches, the introduction of ECT in urology in the nearest future should to be expected.

### 4.1. ECT—Prostate Cancer

New strategies for targeted drug delivery target predominantly cancers specific antigens or use overexpressed enzymes, such as prostate-specific antigen (PSA), to enhance drug penetration [113]. The combination of drug carriers and targeting ligands, which bind to the specific antigens like prostate-specific membrane antigen (PSMA) and prostate stem cell antigen (PSCA) significantly increase the selectivity of cytostatics [114,115]. ECT is an efficient tool for selective drug delivery. However, the available data concerning prostate cancer still derives from a few experimental trials.

In vivo studies on a murine model, ECT has proved its effectiveness on prostate cancer xenografts. Bleomycin was injected into tumor sites 15 minutes before ten pulses of 500 V/cm were delivered. Bleomycin combined with ECT suppressed tumor growth, and 15 days after treatment, xenografts were not detectable [116].

One case report of ECT performed on a patient with ureter-infiltrating prostate cancer proved the feasibility of ECT in the clinic. Due to the high risk of impotence and incontinence, radiotherapy and radical prostatectomy were not feasible. Electrodes were inserted percutaneously through the perineum. The procedure was performed according to the ESOPE protocol. After six months, the MRI showed no sign of tumor persistence or lymphadenopathy. Erection and continence were preserved, indicating the effectiveness and safety of the therapy [78].

### 4.2. ECT—Bladder Cancer

The standard approach in non-muscle-invasive bladder cancer is transurethral resection of bladder cancer (TURB) with following the intravesical installation of cytostatics, usually mitomycin C (MMC) or epirubicin and/or intravesical Bacillus Calmette–Guérin (BCG) [67]. The therapy causes few side effects and is generally efficient for intermediate-risk patients [117]. Nevertheless, therapy failure occurs, and BCG unresponsive high-risk non-muscle-invasive tumors are associated with bad prognosis [118]. Issues such as continuous urine production and relatively short drug residence time limit the therapy effectiveness, thus leading to a higher the relapse rate [119]. There are few different approaches such as the use of hydrogels and mucoadhesive gels, which help to maintain the optimal drug concentration in the bladder [120,121]. Liposomes and nanoparticles can be applied to selectively and efficiently deliver the chemotherapeutics to the tumor site and increase the specificity of photosensitizers used in the photodynamic therapy [122]. Additionally, chemical compounds such as DMSO and hyaluronidase have been shown to upregulate the diffusion and tissue absorption of cytostatics [119]. The physical enhancement of drug delivery in the case of bladder cancer concentrates on microwave-induced hyperthermia and electromotive drug delivery. Both approaches are based on the intravesical placement of the catheter, which can either deliver microwaves to provoke the hypothermia of bladder walls or be the source of electric current, which enhance drug penetration by electrophoresis [123,124]. Although the clinical trials involving the microwave-induced hyperthermia to enhance drug delivery gave promising results, due to insufficient data, the technique is still not commonly used in clinical settings [125,126]. One clinical trial compared the effect of BCG combined with electromotive delivery of mitomycin to the BCG alone [127].

The clinical experience with ECT on bladder cancer remains restricted. One case report described the successful treatment of skin metastases of bladder cancer [128]. The other published studies investigating the in vitro and in vivo effects of ECT with bleomycin or MMC on bladder cancer cell lines and mouse bladder tumors [129,130,131]. Kubota et al. first presented the enhanced uptake of bleomycin in bladder tissue after application of PEFs [131]. Few studies evaluate the outcome of calcium electroporation on bladder cancer in vitro model [132,133]. The in vivo single ECT succeeded in decreasing the tumor growth rate. The further evaluation of MMC in combination with EP in the animal studies showed an improved response to the therapy and prolonged disease-specific survival. The same research results proved the increased effectiveness of cisplatin in ECT on bladder cancer [134].

As mentioned before the combination of TURB with intravesical chemotherapy and/or BCG, although effective, is not always sufficient [135]. For this group of patients, the aim would be to avoid cystectomy, prevent local recurrence, and minimalize the risk of progression to muscle-invasive bladder cancer. Regarding experimental studies, electroporation can be potentially used as a drug delivery approach upregulating the local enhancement of cytostatics inside the bladder wall.

### 4.3. Gene Electrotransfer

**Figure 3:**
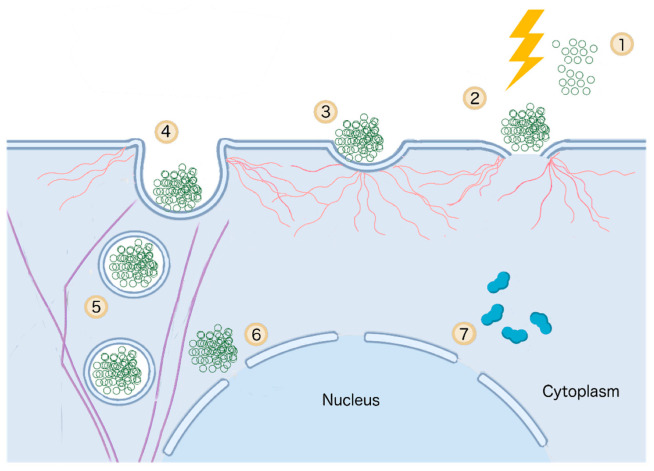
Gene electrotransfer. (1) Plasmid DNA is delivered to the tumor environment; (2) electrical pulses permeabilize the cell membrane; (3) DNA interact with cell membrane on cathode facing site [144]; (4) DNA is inserted into cell membrane with the involvement of actin patches [150]. (5) Plasmids are internalized by endocytosis. Microtubules actively transport DNA molecules through the cytoplasm [136]. (6) DNA leaves endosomes and cross the nuclear envelope [151]. (7) After DNA expression, the proteins are released into the cytoplasm.

EP provides the possibility of non-viral transmission of genes across the cell membrane. Therapeutic use of gene electrotransfer (GET) is focused mainly on two approaches—DNA vaccination and cancer gene therapy [136,137]. The first in vivo GET was carried out in 1991 by Titomirov et al. and since then various experimental attempts were made [138,139,140,141,142]. However, the first clinical application of GET was performed by Daud et al. in 2008 for transfer of interleukin-12 in patients with metastatic melanoma [143]. The mechanism of electroporation-driven gene transfer is a multi-step process. Once the membrane becomes permeable, the cell membrane in the regions opposite to cathode interact with the DNA [144]. The dynamics of that process depend on PEF features such as polarity, repetition, frequency, and duration [145]. It was observed that the application of nanosecond pulses in combination with microsecond or millisecond electric pulses can result in an increase in DNA transfection rate in the targeted cell [146]. DNA diffusion across the membrane takes minutes to hours [144]. The research suggests that the model of endocytosis explains the process of transport of DNA aggregates [147,148]. At first, the electric-field-independent formation of DNA-membrane complex occurs. The aggregation is size-dependent, meaning that the helices containing more than 25 base pairs (bp) aggregate, which cannot be observed for 15 bp helices [149]. The number of small DNA molecules, so-called free DNA, can be directly transported into the cytoplasm [149]. After the aggregation, electropore is formed by the application of PEF, which results in the movement of the genetic material directly to the cytoplasm. The amount of translocated genetic material decreases with the size of the nucleic acid [149]. While crossing the membrane, plasmid interacts with the hydrophilic head groups of membrane phospholipids and cell cytoskeleton [150]. Successful DNA electrotransfer involves the migration of the DNA to the nucleus. DNA may cross the nuclear envelope during the cell division or be actively transported by the nuclear pore complex [151]. The current understanding of the electrotransfer mechanism was reviewed by Rosazza et al. [136] (Figure 3).

GET is commonly performed on muscle or skin tissue. Due to the immunological function and non-restricted access, the skin remains one of the natural candidates for electrotransfer [152]. In general, therapy on the skin is effective. The method found its application in DNA vaccines, skin ischemia treatment, and the stimulation of wound healing [153,154,155,156,157]. GET to muscle provides a high expression of transferred genes for a relatively long period of time and enables the efficient production of transcription factors [158]. The heterogenicity of the cancer tissue makes the transfection directly into the tumor complicated. However, it is possible and effective when the electroporation-based method is properly optimized. [158]. Numerous in vivo and in vitro studies prove the efficiency of GET in gene transfer to tumors [146,159,160,161,162,163].

Cancer gene therapy DNA encoding immunomodulatory factors and cytokines constitute the possible choice for anti-cancer gene transfection [164]. The efficiency of electroporation-based in vitro transfection oscillates around 80%, which is comparable to the standard viral transfection. The electroporated genetic construct does not typically integrate with the genome. Therefore, it is considered safer than other transfection methods [165]. The solution to the lack of integration can be provided by GET of CRISPR/Cas9 nuclease system. CRISP-Cas9 mediated genome editing enables the specific integration of electrotransferred plasmids with the genome in vivo [166].

Apart from the delivery site, the effectiveness of GET depends on length, voltage, number of pulses, kind of used electrodes, and interval time between the injection and pulse delivery [167]. The electric field intensity has to be low enough to ensure the survival of cells. DNA used in GET targeted against tumor generally consists of plasmids encoding cytokines that modulate the tumor and growth factors that induce the antiangiogenic effect in the tumor microenvironment [168,169,170,171]. The efficiency of GET was investigated on urological cancers. Since the approach is relatively new, and efficiency strongly depends on pulse parameters, most of the experiments were carried out on animal models. The majority of published trials of GET on urological malignances use plasmids encoding specific tumor antigens. Several strategies of DNA electrotransfer targeted against prostate cancer have been examined in clinical trials.

### 4.4. GET—Prostate Cancer

DNA-based cancer vaccines have proven their efficiency to induce cytotoxic T lymphocytes specific for prostate antigens. Among antigens of prostate cancer, PSA, PSMA, and PSCA are primary candidates applied in immune therapy [172].

Electroporation has been studied as a possible approach for delivering DNA vaccines to prostate cancer in animal models. *PSA* GET proved to be effective. Nevertheless, the parameters needed to be optimized to achieve both the high efficiency of transfection and immune response against antigens. It appeared that the combination of high short and low long pulses were the most effective [173]. In another two in vivo experiments, plasmids encoding PSA and PSCA antigens were delivered by intramuscular electroporation [174,175]. GET resulted in significant production of IFN-γ, which generated a high level of Th-1 T-cell. The therapy provided the reduction of a tumor mass [175]. In another in vivo experiment, the *p53* mRNA GET achieved the remarkable suppression of prostate tumor growth [176].

In a clinical phase I/II trial on patients with prostate cancer, the plasmid encoding epitope from PSMA was transfected by the electroporation of muscle tissue. The domain of fragment C of tetanus toxin was added to the injected plasmid to upregulate the immune response. Electroporation appeared to be acceptable by patients, although it was painful for a short time. No additional risks or safety concerns connected with electroporation were identified. The response to the therapy was remarkably higher among patients who received DNA injection combined with EP comparing to the control group, where EP did not follow DNA vaccination [79]. The other phase I clinical study evaluated the feasibility of a PSA antigen vaccine combined with intradermal electroporation. Prior to the vaccination, the androgen deprivation therapy (ADT) was performed on patients to induce T-cell infiltration. The electrotransfer successfully increased PSA-specific CD8+ T-cell mediated immune reaction in patients where ADT primarily triggered the reaction. Moreover, the same effect has been obtained in case of patients where the ADT failed to prime T-cells. The electrotransfer was easy to perform, well-tolerated, and show no severe side effects [80]. A phase I clinical trial evaluating the electrotransfer of DNA Vaccine together with checkpoint inhibitors and PROSTVAC for prostate cancer is currently on-going (NCT03532217).

### 4.5. GET—Bladder Cancer

The primary trials performed on animal models showed that electroporation is an effective method of gene transfection to submucosal interstitial cells and urothelial cells [177]. Subsequently, two in vivo trials evaluated the effectiveness of GET with the genomic DNA of recombinant bacillus Calmette-Guerin (BCG) with IL-12 [178,179]. Both studies confirm that DNA electrotransfer increased INF-γ and IL-12 secretion inducing infiltration of CD4+/CD8+T-cells and NK cells into the tumor site. Local immune reaction inhibited tumor growth, resulting in significantly prolonged cumulative survival of mice treated with poly-rBCG and mIL-12. The results of both studies suggest that BCG DNA electrotransfer with IL-12 instead of intact live BCG can be effectively applied to stimulate the immune reaction against bladder cancer [180].

### 4.6. GET—Renal Cancer

The effect of *tumor necrosis factor-related apoptosis-inducing ligand* (*TRAIL*) cDNA plasmid electrotransfer was investigated on the murine model. DNA plasmid injection and electroporation were introduced directly to the tumor. The study shows that *TRAIL* gene therapy induced growth suppression and apoptosis of renal cancer tumor cells. No severe side effects were reported. Moreover, the application of *TRAIL* electrotransfer combined with 5-FU enhanced the outcome of the therapy. The study proved that *TRAIL* GET is a safe and effective treatment method, but further studies are needed to evaluate the feasibility of this approach on humans [181]. Another study on the murine model evaluated the possibility of electrotransfer of plasmids encoding IL-12 for renal cancer treatment. The therapy resulted in significant inhibition of tumor growth after *Il-12* GET compared to GET of empty plasmids [182].

### 4.7. GET—Adoptive Transfer of Autologous T-Cells

Today, electroporation-based techniques are also used to modulate the immune system to target solid urological tumors or metastatic malignancies. GET can be utilized for the transport of genetic material, encoding the chimeric receptor or specific antigen into immune-competent cells. Modulation of the immune response includes the genetic modification of the immune-competent cells, like the production of chimeric antigen receptor (CAR) T cells, CAR-NK cells, or even the stimulation of NK cells proliferation and killing properties—cytokine-induced killer cells (CIK cells) [183,184,185]. The advantages of the application of electroporation in CAR-T technology are a simple manufacturing procedure and have a relatively low cost when compared to other methods [165]. Currently, several attempts of CAR-T therapy were successful in treating urological malignancies. However, it has to be emphasized that none of the further mentioned trials concerning CAR-T for urological malignancies applies electroporation for gene transfection. Generally, due to the high expression on the prostate gland tissue, PSMA and PSCA are utilized as the targets for the CAR-T cell therapy for prostate cancer [186,187,188]. High effectivity of the therapy led to the registration of CAR-T against PSMA for phase I clinical trials in patients with metastatic prostate cancer (NCT01140373). Besides, PSCA-CAR-T Cells undergo phase I clinical trials for metastatic PSCA+ castration-resistant prostate cancer (NCT03873805). The therapy seems to be promising for other urological malignancies as well. For instance, EGFR-specific CAR-NK-92 cells were proved in vitro to exhibit a synergistic effect with cabozantinib against renal cell carcinoma [189]. The efficiency of NK cells against the metastatic renal tumor is being evaluated in clinical trials (NCT02843607). Considering bladder cancer, the CAR-T therapy against urothelial tumor undergoes the clinical trial Phase I/II (NCT03185468). Although viral vectors remain the most often used in CAR-T manufacturing, electroporation seems to be a reasonable and promising alternative, which hopefully can be introduced to urology.

## 5. Conclusions

The hallmark of EP is the diversity of applications. Depending on the pulse parameters, it can be involved in a wide range of treatments. Considering the focal ablation, IRE has already proven to be the feasible, safe, and effective method of urology cancer treatment. The improved algorithms and the methods of electric field distribution monitoring enable precise ECT and IRE. The need for general anesthesia and muscle relaxation can be overcome by optimal pulse parameters. In the future, electroporation trials should involve the application of immune-stimulating agents and the performance of repetitive therapies. However, no long-term outcome evaluated in a randomized trial restricts the clinical application of electroporation in urology. As previously mentioned, with optimized parameters, GET constitutes a reasonable alternative to the gen vectors in immune therapy. The in vitro efficiency of GET can be compared with viral transfection. The protocols enabling the GET to tumor sites are developing, and studies provide promising results [190]. Prospectively, GET in urology might be applied not only in oncology but also to treat bladder dysfunctions and erectile dysfunction [191]. Thinking globally, the relatively low cost of EP enables less wealthy countries to have access to the therapy [106]. The generator of electric impulses and electrodes can be produced at reasonable prices. Moreover, in the case of IRE, no additional drugs are needed. The cytostatics administrated in ECT can be substituted with already registered and widely available calcium chloride, with similar results [106]. Decisively, the elucidation of the EP potential, in presented approaches in urology, will keep scientists busy for a long time.

## Figures and Tables

**Table 1:** Clinical trials of IRE for small renal masses.

Type of Trial	Patients Number	Short Description	Study Outcome	Renal Function	Complications	Therapy Protocol	Ref.
Retrospective	20	IRE for focal treatment of cT1a renal cancer	initial treatment success rate of 90% (18/20). 17% (1/6) recurrence rate during a one-year follow-up	no significant differences in creatinine levels before and 6 weeks after IRE	3/20 urinary retention, 2/20 perinephric hematomas, 2/20 patients suffered from pain	3–4 electrodes, for tumors over 2.5 cm, multiple probes, 30–40 A, pulse duration 100 µs at 1 Hz, 140 pulses with electrode polarity change after 70 pulses	Trimmer et al. [65]
Prospective	42	IRE for focal treatment of cT1a renal cancer	initial treatment success rate of 93% (39/42), 83% 2-year local recurrence-free survival rate	pre and post-operative glomerular filtration rate of patients did not differ significantly	2/42 urinary retention, 4/42 perinephric hematomas, 1/42 patient suffered from pain	3–4 electrodes, for tumors over 2.5 cm, multiple probes 2000 V/cm, 100 µs at 1 Hz, 140 pulses with electrode polarity change after 70 pulses	Canvasser et al. [66]
Prospective	10	IRE for focal treatment of cT1a renal cancer	10% (1/10) recurrence rate during 6 months of follow-up	no significant differences in creatinine levels in pre-IRE tests and 1 week, 3 months, 6 months, and 12 months post-IRE tests	1/10 perinephric hematoma and pyelonephritis with fever, 1/10 blood clot in the ureter, 1/10 painful micturition, 1/10 hematuria	6 electrodes, active tip exposure 10–25 mm, 20–40 A, 100 pulses, 90 µs	Mara et al. [64]

**Table 2:** Clinical trials of IRE, electrochemotherapy (ECT), and gene electrotransfer (GET) for prostate cancer.

Therapy	Type of Trial	Patients Number	Short Description	Urinary Continence	Erectile Function	Study Outcome	Therapy Protocol	Ref.
IRE	Retrospective	429	IRE for focally (123), sub-whole-gland (154), whole-gland (134) or for recurrent prostate cancer (63) after previous radical prostatectomy or radiation therapy,	IPSS-Score (urinary symptoms)- 72.8% of patients with no change or improvement; 27.2% with a decrease during the follow-up period (about 12 months)	IIEF-5 score–14/124 (11.3%) patients developed erectile dysfunction after IRE in 4/124 (3%) patients dysfunction persisted longer than one year	47/429 (10.9%) of patients with prostate cancer recurrence in 72 months follow-up (27/429 in-field and 20/429 out-of-field recurrence)	5 ± 1 electrodes; 1518.13 ± 204.05 V/cm	Guenther et al. [73]
IRE	Prospective	123	IRE for prostate gland ablation as a primary procedure; largest reported cohort of patients treated with IRE.	80/81 (98.8%) patients remained pad free and 70/75 (93.3%) remained leak-free at 12 months after treatment.	40/53 (76%) patients with normal erectile function, 9/53 (17%) with decreased, but enough for sexual activity, and 4/53 (7%) with total erectile dysfunction 12 months after treatment	23/102 (22.5%) of patients with prostate cancer recurrence 12 months after treatment (10/102 (9.8%) in-field recurrence. 13/102 (12.7%) out-of-field recurrence)	90 pulses; 1500 V/cm; 70 µs or 90 µs; 5 mm distance from vital structures; safety margin of 5 mm to 10 mm from the targeted area;	Blazevski et al. [74]
IRE (H-FIRE)	Prospective	40	H-FIRE for prostate gland ablation, as a primary procedure 8/40 patients underwent prostatectomy four weeks after treatment	40/40 (100%) patients remained pad free	14/14 (100%) patients with normal erectile function	high-frequency bipolar pulses can be safely applied for IRE of prostate cancer; oncological outcome was not evaluated	HF bipolar pulses; 250 bursts; 50 pulses in one burst; 1 burst/second; 10-s inter-burst delay; 2–6 needle electrodes; interelectrode distance <20 mm	Dong et al. [75]
IRE	Prospective	18	IRE for localized radio-recurrent prostate cancer,	8/11 (72.2%) of patients remained pad-free at six months; 10/11 (90.9%) pad-free at 12 months	2/6 (33.3%) patients with normal erectile function 6 months after treatment and 2/4 (50%) patients with normal erectile function 12 months after treatment (One patient recovered at 12 months from erectile dysfunction); a significant decrease in EPIC sexual score between baseline and six months	2/10 (20%) of patients with prostate cancer recurrence 12 months after treatment (1/10 (10%) in-field recurrence and 1/10 (10%) out-of-field recurrence)	90 pulses; 1500 V/cm; pulse; 70 µs or 90 µs; safety margin of 5 mm to 10 mm from the targeted area; active tip length 10-20 mm; interelectrode distance 7-19 mm	Scheltema et al. [76]
IRE	Prospective	63	IRE for prostate gland ablation, as a primary procedure	44/45 (98%) of patients remained pad-free at 6 months 45/45 (100%) pad-free at 12 months.	8/26 (31%) patients with erectile dysfunction at 6 months after IRE and 3/13 (23%) with erectile dysfunction 12 months after IRE.; the significant difference in EPIC sexual score between baseline and 6 months after IRE	11/48 (22.9%) of patients with prostate cancer recurrence 12 months after treatment (7/48 (14.6%) in-field recurrence and 4/48 (8.3%) out-of-field recurrence)	90 pulses; 1500 V/cm; 70 µs or 90 µs; safety margins of 5 mm or 10 mm from the targeted area; 5 mm distance from vital structures	Van Den Bos et al. [77]
ECT	Case report	1	ECT for recurrence of prostate cancer by a 67-year-old patient	IPSS—no significant impairment after six months (remaining mild incontinence)	IIEF-5 score restored to the pretreatment level six months after ECT (remaining mild erectile dysfunction)	MRI six months after the treatment, showed no evidence of tumor persistence	8 min before electroporation administration of 29 mg of bleomycin i.v.; eight pulses; 100 µs; 4 Hz frequency; 1642 ± 812 V	Klein et al. [78]
GET	Prospective, two-arm	30	Patients with, biochemically recurrent prostate cancer (rising PSA but no radiological evidence of disease) received an intramuscular injection of DNA encoding PSMA+DOM C or injection followed by EP	Not evaluated	Not evaluated	Vaccination alone and with EP was well tolerated; vaccination alone showed 1.7 increase of anti-DOM IgG; vaccination with EP showed a 24.5-fold increase of anti-DOM IgG compering to baseline; responses persisted up to 18 months of follow-up	Vaccinations were administered at 0, 4, and 8 weeks, with booster doses at 24 and 48 weeks; EP was performed by two needles, which after injection served as electrodes; five pulses; 20 µs; 8.3 Hz frequency; 8 mm intra-needle distance	Low et al. [79]
GET	Prospective	15	Patients with biochemically recurrent prostate cancer without macroscopic disease received an intradermal injection of DNA encoding PSA followed by EP	Not evaluated	Not evaluated	Intradermal vaccination with skin EP is feasible; only minor discomfort occurred; vaccination with EP enhanced pre-existing or boosted PSA-specific immunity	Vaccination was administrated every four weeks for five months; EP was applied immediately after each injection with two parallel rows of needles (6 needles/row); 10 pulses: 2 (1125 V/cm; 0.05 µs) and 8 (275 V/cm; 10 µs)	Eriksson et al. [80]
